# EGFR TKI as first-line treatment for patients with advanced EGFR mutation-positive non-small-cell lung cancer

**DOI:** 10.18632/oncotarget.20095

**Published:** 2017-08-09

**Authors:** Xueli Nan, Chao Xie, Xueyan Yu, Jie Liu

**Affiliations:** ^1^ School of Medicine and Life Sciences, University of Ji’nan-Shandong Academy of Medical Sciences, Shandong, China; ^2^ Department of Oncology, Shandong Cancer Hospital Affiliated to Shandong University, Shandong, China; ^3^ Department of Oncology, Shandong Provincial Chest Hospital, Shandong, China; ^4^ Shandong Academy of Medical Sciences, Shandong, China

**Keywords:** EGFR TKI, first-line treatment, non-small-cell-lung cancer, EGFR mutations, combined therapy

## Abstract

After the discovery of activating mutations in EGFR, EGFR tyrosine kinase inhibitors (TKIs) have been introduced into the first-line treatment of non-small-cell lung cancer (NSCLC). A series of studies have shown that EGFR TKI monotherapy as first-line treatment can benefit NSCLC patients harbouring EGFR mutations. Besides, combination strategies based on EGFR TKIs in the first line treatment have also been proved to delay the occurrence of resistance. In this review, we summarize the scientific literature and evidence of EGFR TKIs as first-line therapy from the first-generation EGFR TKIs to conceptually proposed fourth-generation EGFR TKI, and also recommend the application of monotherapy and combination therapies of the EGFR-based targeted therapy with other agents such as chemotherapy, anti-angiogenic drugs and immunecheckpoint inhibitors.

## INTRODUCTION

Non-small-cell lung cancer (NSCLC) is believed as one of the main reasons that cause deaths from cancer worldwide. According to statistics, the quantity of new lung cancer is expected to reach 222,500 in the United States in 2017 [1]. Before introducing genomic epidermal growth factor receptor (EGFR) inhibitor therapy, despite platinum-based combination chemotherapy, the overall survival (OS) of most patients with advanced non-small-cell lung cancer (NSCLC) is less than one year [2]. With the deep understanding of EGFR gene mutations, the treatment of NSCLC has entered an era of co-directed therapy by histology and genotyping, which allows patients to obtain personalized molecular targeted therapies [3].

The HER family includes EGFR, HER2 (ErbB2), HER (ErbB3) and HER4 (ErbB4). Through combining with its ligands, the EGFR takes shape dimers with other EGFR or other HER family members and experiences autophosphorylation at the crucial tyrosine residues, leading to activation of some downstream signalling pathways, which mediates a variety of cellular processes, including proliferation, survival and apoptosis. EGFR signaling can be constitutively activate through genic mutation or genic amplification or both, which has been shown to be closely related to the occurrence, progression and poor prognosis of NSCLC [4–6]. Besides, there have studies demonstrated activation mutations in the tyrosine kinase domain of EGFR and this percentage of mutations may range from 15% to 40%, particularly common in Asian, female, never or light smoking NSCLC [7–10]. More than 90% of the known activating EGFR mutations are deletion of exon 19 (in-frame) and point mutation of exon 21 (L858R) [4, 5]. Subsequent clinical studies have demonstrated that these two mutations have different susceptibilities to TKIs treatment [11–13].

After the discovery of the EGFR gene, EGFR tyrosine kinase inhibitors (TKIs) have also been published one after another. EGFR TKIs have been recommend by the American Society of Clinical Oncology (ASCO), the European Society for Medical Oncology (ESMO), and the National Comprehensive Cancer Network (NCCN) as a potential first-line treatment for advanced NSCLC patients with EGFR mutations positive. Recently, a considerable number of clinical studies are designed to assess the efficacy and safety of different types and different treatment modalities of EGFR TKIs as first-line treatment for NSCLC. In this review, our goal is to help clinicians make decisions in consideration of existing evidences and provide forthcoming methods for patients harboring EGFR mutations.

### EGFR TKI monotherapy

EGFR TKI monotherapy has become the recommended treatment strategy and the cornerstone of combined therapy for NSCLC patients harboring EGFR mutation*.*

#### EGFR TKI versus chemotherapy

Compared with traditional platinum-based combination chemotherapy, EGFR TKI unprecedentedly brought a better clinical benefit and quickly laid its own position in treating advanced non-small cell lung cancer patients with EGFR mutation.

#### First-generation EGFR TKIs

The working mechanism of first-generation EGFR-TKIs is to block the activation of downstream signaling induced by EGFR through binding to the ATP-binding sites.

#### Gefitinib

Gefitinib is considered as an oral first-generation EGFR TKI. In 2015, gefitinib was approved by USFDA as a first-line treatment for metastatic NSCLC patients with activating EGFR mutations [14]. There are four major studies supporting gefitinib as first-line therapy for NSCLC harboring EGFR mutations [15–18]. The IPASS (Iressa Pan-Asia Study) [15] was regarded as the milestone for the clinical application of EGFR TKI, because it was the first randomized clinical trial to compare EGFR-TKI with chemotherapy at first-line treatment for advanced NSCLC with adenocarcinoma. The final results reported that improved progression-free survival (PFS) was inferior in the chemotherapy arm than the gefitinib arm. However, the overall survival (OS) was no difference comparing the overall population with EGFR mutation-positive patients [18.8 months vs. 17.4 months hazard ratio (HR), 0.90; 95% confidence intervals (CI), 0.79 to 1.02; *P* < 0.109]. Interestingly, the PFS benefit only existed in the EGFR mutation positive subgroup (median 9.5 vs. 6.3 months, HR 0.48 [0.36–0.64], *p* < 0.001) not in the EGFR mutation negative subgroup (median 1.5 vs. 5.5 months, HR 2.85 [2.05–3.98], *p* < 0.001) than chemotherapy . The final finding considered that EGFR mutation could predict the response to gefitinib, so it may be the most promising predictive biomarker. The outcomes of First-SIGNAL, NEJ002 and WJTOG3405 trials [16–18] were same to those of IPASS in NSCLC patients carrying positive EGFR mutation through comparing gefitinib with doublet chemotherapy as first-line therapy. In these studies, rash, diarrhea and liver dysfunction were the most common adverse events (AEs) reported with gefitinib. In addition, interstitial lung disease was rare but fatal. However, the longer PFS did not turn into OS advantage—the reason behind this failure could be the result of a cross-over effect. Moreover, these studies established EGFR mutation status can replace clinical predictors to become more valuable predictive factor and recommended gefitinib as the first-line treatment for NSCLC with EGFR mutation.

#### Erlotinib

Erlotinib is an reversibly oral EGFR-TKI and it received approval from FDA as a first-line treatment for patients with EGFR mutation in 2013. Be different from gefitinib in Asian population, EURTAC [19] was the primary trial to prove the Caucasian population can also benefit from EGFR TKI as first-line treatment. In the present study, the researchers randomized 173 NSCLC patients with EGFR mutations to erlotinib and chemotherapy. The median PFS (primary endpoint) was 9·7 months in erlotinib set longer than 5·2 months in chemotherapy set at time cut-off (HR 0·37, 95% CI 0·25−0·54; *p* < 0·0001). Similar to gefitinib, OS was no significant difference between two groups, too. The data was 22.9 months in EGFR TKI block in comparison with 18.8months in the chemotherapy block (HR 0.80; *P* = 0.42). The most commonly reported adverse events in the erlotinib group were rash and increased aminotransferase concentrations. Another randomized, phase 3 study (OPTIMAL) [20, 21] undertaken in China also confirmed the superiority of erlotinib than first-line chemotherapy in NSCLC patients harboring EGFR mutation (13·1 vs 4·6 months; HR 0·16, 95% CI 0·10–0·26; *p* < 0·0001)

In the 15th World Congress of Lung Cancer, Professor Wu reported the results of ENSURE study in which 217 Asian patients randomly received erlotinib (150mg/d ) or gemcitabine plus cisplatin. There was no doubt that the median PFS (primary endpoint) for erlotinib was superior to the chemotherapy (11.0 and 5.5 months, respectively, HR = 0.33,*P* < 0.0001). Interestingly, the PFS of patients treated with erlotinib was 11.1 months and 8.3 months in the 19 exon deletion subgroups and L858R mutation in different subgroup, respectively; while it was only 4.3 months and 5.8 months in corresponding chemotherapy arm, indicating that the two types of EGFR mutations treated with erlotinib have significant benefits, and 19 exon deletion benefits more.

#### Icotinib

Icotinib is an oral, selective EGFR TKI made in China [22]. A randomized, phase III study (CONVINCE ) [23] proved that icotinib made significant improvements of PFS in comparison with the chemotherapy set (296 days [95% CI 255–355] vs 219 days [189–253]; HR 0.67, 95% CI 0.49–0.90, *p* = 0.008) . In addition, superior tumor response rate and safety were inclined to icotinib (64.8% vs 33.8%, *p* < 0.001; AEs in icotinib set,104 [70.3%] vs 121 [88.3%], *p* < 0.001). Elevated transaminase (29.1%), rash (17.6%) and diarrhea (9.5%) were the most common AEs in patients treated with icotinib. Studies about first- and second-genetation EGFR TKI as first-line treatment for EGFR mutation patients were showed in Table 1.

**Table 1:** Studies of first- or second-generation EGFR TKI in treated-naive patients with lung adenocarcinoma

Study	EGFR TKI	Chemotherapy	Mutation	Median PFS (months)	ORR (%)	Median OS (months)
IPASS	Gefitinib	Carboplatin + paclitaxel	All	9.5 vs 6.3	71.2 vs 47.3	21.6 vs 21.9
WJTOG 3405	Gefitinib	Cisplatin + docetaxel	mEtGFR	9.2 vs 6.3	62.1 vs 32.2	36 vs 39
First-SIGNA	Gefitinib	Cisplatin + gemcitabine	All	5.8 vs 6.4	55.4 vs 46.0	22.3 vs 22.9
			mEGFR subgroup	8.0 vs 6.3	84.6 vs 37.5	27.2 vs 25.6
NEJ002	Gefitinib	Carboplatin + paclitaxel	mEGFR	10.8 vs 5.4	73.7 vs 30.7	27.7 vs 26.6
EURTAC	Erlotinib	Cisplatin + docetaxel	mEGFR	9.7 vs 5.2	64 vs 18	19.3 vs 19.5
OPTIMAL	Erlotinib	Gemcitabine + carboplatin	mEGFR	13.1 vs 4.6	83 vs 36	NA
Lux-lung 3	Afatinib	Cisplatin + pemetrexed	all	11.1 vs 6.9	56.1 vs 22.6	28.2 vs 28.2
			mEGFR	13.6 vs 6.9		
Lux-lung 6	Afatinib	Gemcitabine + cisplatin	mEGFR	11.0 vs 5.6	66.9 vs 23.0	23.1 vs 23.5

PFS = Progression Free Survival.

ORR = Objective Response Rate.

OS = Overall Survival.

mEGFR = EGFR mutation.

NA = Not Available.

### Second-generation EGFR TKIs

The development of second-generation EGFR TKIs is to overcome the acquired resistance which comes from the failure of first-generation EGFR TKIs. So the working mechanisms of second-generation EGFR TKIs are not exactly similar to first-generation EGFR TKIs.

#### Afatinib

Afatinib is a novel, orally bioavailable irreversible EGFR inhibitor, which is an aniline-quinazoline derivative with a reactive acrylamide group that can modify conserved cysteine residues by Michael addition within the catalytic domains of EGFR, HER2 and ErbB-4. By irreversible covalent bond, it blocks enzymatic activity of active ErbB receptor family members. Because the irreversible covalent binding can inhibit the kinase activity until the synthesis of new receptors, the action time of afatinib may longer than reversible EGFR TKIs [7].

LUX-Lung 3 [11] trial was a global, randomized study which evaluated the efficacy and safety between afatinib and chemotherapy as a first-line treatment in patients with proven EGFR mutation and lung adenocarcinoma histologically. Eligible patients (*n* = 345) were randomly allocated in 2:1 ratio to either afatinib arm or pemetrexed plus cisplatin [24]. Generally, afatinib showed significant prolongation of median PFS than chemotherapy and median PFS was 11.1 and 6.9 months, respectively (HR: 0.58; 95% CI: 0.43–0.78; *p* = 0.0004). In addition, the median PFS was 13.6 months for patients treated with afatinib in comparison with 6.9 months for patients treated with chemotherapy in Del 19/L858R subgroup (HR: 0.47; 95% CI: 0.34–0.65; *p* < 0.0001). Furthermore, the RR was also significantly higher in afatinib group compared with in chemotherapy group (56.1% and 22.6%, respectively).

The largest prospective phase III study (LUX-Lung 6) [12] was undertaken in EGFR mutation-positive lung cancer. Researchers randomized 364 patients to receive afatinib or gemcitabine plus cisplatinin in a 2:1 ratio. Compared with chemotherapy, in patients with common EGFR mutations (Del19/L858R), mPFS was significantly prolonged with afatinib (11.0 vs 5.6 months; HR: 0.28; *p* < 0.0001). The afatinib group also achieved higher objective response (66.9 vs 23.0%; *p* < 0.0001) and disease control rates (92.6 vs 76.2%; *p* < 0.0001) .

A combination analysis of LUX-lung3 and LUX-lung6 [13, 25] had proved that 19 exon deletion tumours were different from L858R mutations tumours, and the former may be better: median OS was better in the 19 exon deletion compared with the chemotherapy group no matter in LUX-Lung 3 or LUX-Lung 6, while no statistically significant difference was observed in the L858R mutations group. The most common treatment-related AEs were diarrhea , rash/acne, stomatitis and nail effects.

Compared with first—generation EGFR-TKIs, afatinib is the only one to exhibit an OS benefit in patients harboring 19 exon deletion instead of L858R mutations than chemotherapy. This benefit may be attributed to the following reasons: distinct biological properties of 19 exon deletion and L858R mutations, the differences existing in reversible EGFR TKI and the irreversible ERBB family blocker afatinib, or both. The above results suggested that clinicians should make a difference between patients harbouring 19 exon deletion L858R mutations in the stratified analysis of future clinical trials. But in current state, all those patients carrying EGFR mutations still should be treated with EGFR inhibitors as first-line no matter first-generation or afatinib based on better PFS.

#### Dacomitinib

Dacomitinib as an pan-HER TKI is to target EGFR, ErbB2 and ErbB4 kinase, and it domains of the EGFR signaling pathway by irreversibly bind the ATP [26, 27]. It is published that the PFS (the primary endpoint) demonstrated a statistical improvement for dacomitinib over erlotinib as second-line (median 2.8 vs 1.9 months, *p* = 0.012) [28]. In a phase II study, dacomitinib was evaluated as first-line treatment for patients with either EGFR mutation or less than a 10 pack-year smoking history. Interim results demonstrated that the primary endpoint of PFS at 4 months was 96% in overall population. In addition, the patients with EGFR mutation-positive gained an objective responses of 74% (95% CI: 59–86) [29].

### Third-generation EGFR TKIs

Nowadays, several EGFR TKIs such as gefitinib, erlotinib and afatinib are approved worldwide for the treatment of NSCLC harbouring EGFR mutations. These target drugs can improve PFS, ORR and quality of life in comparison with conventional chemotherapy in first-line setting. Unfortunately, all patients treated with gefitinib, erlotinib or afatinib will unavoidably generate resistance to previous drug [30]. To a large extent, either acquired or intrinsic resistance can reduce the efficacy of EGFR TKIs [31]. These published mechanisms include the development of T790M mutation, CMET amplification, HER2 amplification, histological transformation to small-cell histology, and so on [32, 33]. Among them, the most important mechanism of acquired resistance is the EGFR T790M mutation. It was reported that occurrence frequency of T790M mutation was from 49% to 63% after rebiopsies [34–36]. The competitive combination ability of first-generation and second-generation TKIs has been reduced by T790M mutation through changing the affinity of EGFR for ATP [31, 37]. To overcome this new difficulty, different third-generation EGFR-mutant selective TKIs, such as osimertinib, rociletinib and olmutinib, came into being (Table 2) [38, 39]. These agents are specifically designed to inhibit EGFR T790M without wild-type EGFR, which structurally different from first-generation and second-generation inhibitors [40, 41].

**Table 2:** The clinical studies for third-generation EGFR TKI

	Study	Number^a^	ORR	PFS (months)
AZD9291	AURA1 I	253	51% T790 m+ 60% T790 m– 28%	NA
AZD9291	AURA2 I/II	472/210	71%	8.6
AZD9291	AURA3 tIII	419/279	71%	10.1
CO1686	I/II	612/69	T790 m+ 45% T790 m– 17%	6.1 1.8
HM61713	I/II	71	56%	7.0

^a^Number of patients assigned/number of patients treated by third-generation EGFR TKI.

ORR = Objective Response Rate.

PFS = Progression Free Survival.

NA = Not Available.

#### Osimertinib (taggriso, AZD9291)

Osimertinib could irreversibly and selectively inhibit both T790M mutation-positive EGFR and sensitizing EGFR [42, 43]. In recent decades, a global phase 1 trial (AURA 1) [44] reported that the ORR of osimertinib was amazingly up to 51% in all patients (95% CI: 45%–58%). The ORR was 61% (95% CI: 52%–70%) and 21% (95% CI: 12%–34%) in patients with EGFR T790M mutations and without the EGFR T790M mutations, respectively. The AURA2 Phase I/II study [44] convinced that patients with activating EGFR mutations and T790M mutations can benefit from an 80 mg once-daily dose of osimertinib at second-line treatment. The final ORR was 71% and animmature PFS was 8.6 months. Recently, the New England Journal published preliminary results of the AURA3 study [45] in which osimertinib significantly improved the mPFS compared with chemotherapy for patients carrying EGFR T790M and previously treated with EGFR TKIs (10.1 vs 4.4 months HR = 0.3, 95% CI 0.23–0.41, *P* < 0.001). Besides, objective response rate of 71% also supports osimertinib. In addition, two phase I expansion cohorts were designed to assess that osimertinib was used for first-line treatment for EGFR mutation-positive advanced NSCLC**.** In this study, 60 treatment-naïve patients received two dose levels (80 mg; 160 mg). The end ORR was 77% (95% CI 64, 87) and the 160 mg cohort got the higher ORR than the 80 mg cohort (87% versus 67%). The mPFS was 19.3 months (95% CI 13.7, NC) among all patients, and the mPFS of 80mg cohort didn’t reach (95% CI 12.3, NC) while the other cohort was 19.3 months (95% CI 11.1, 19.3) at the time cut-off. Also, the disease control rate reached up to 97% (95% CI 88.5, 99.6). [46] The most common adverse events of osimertinib were diarrhea, rash, nausea, and decreased appetite.

#### CO1686 (rociletinib)

Similar to osimertinib, CO1686 is considered as another prospective third-generation irreversible EGFR TKI [41]. In primary phase I/II trial, 130 patients (but the study ultimately included 612 patients) were randomly assigned to take the free-base form of rociletinib or the hydrogen bromide salt form. The update data showed median PFS of the T790M-positive set and T790M negative was 6.1 months (95% CI, 4.2 to 9.6) and 1.8 months (95% CI, 1.2 to 3.0), respectively. The final response rate was 45% in patients with T790M+. The common adverse events were nausea, fatigue, diarrhea, and grade 3 hyperglycemia. The updated data showed the response rate of rociletinib from initial 59% to a confirmed 45% among patients with T790M+. The researchers attributed this reduction to a fact that more than half patients had performed only once imaging evaluation at data cutoff. Besides, the optimal dose of rociletinib, 500 mg or 625 mg bid, is still not clear [38, 47]. So it is necessary to carry out more clinical studies to assess rociletinib before large-scale applications.

#### HM61713 (olmutinib)

HM61713 is a new, oral inhibitor can target selectively either activating EGFR mutations or T790M-disease, but except EGFR wild-type. An ongoing phase I/II trial of HM61713 was performed in advanced NSCLC patients who had failed in previous treatment. The preliminary data reported that median PFS was 7.0 months (95% CI 5.5–8.3) and there were 26 (34%) patients still being treated at data cut-off. Diarrhea, rash, pruritus, and nausea were most frequent adverse reactions. Among 71 patients evaluated for remission, 40 (56%) reached an objective response by researchers examination (31 [44%] confirmed) and median duration of response was 8.3 months (range 5.6–NE). It is encouraging that disease control rate reached 90% [48].

#### AC0010

AC0010 is an irreversible EGFR inhibitor based on pyrrolopyrimidine, which is structurally different from previously published pyrimidine-based irreversible EGFR inhibitors, such as osimertinib and rociletinib. In a xenograftmodel, AC0010 caused tumors complete remission more than 143 days without weight loss in EGFR-active and T790M mutations. Metabolite detection results demonstrated that AC0010 didn’t target wild-type EGFR and no off-target effects occurred. The safe dose of AC0010 range from 50 to 550 mg daily in NSCLC patients, and no severe adverse events were reported. Besides, the investigators observed the objective responses happen to patients with EGFR T790M mutation [49].

Briefly, the third generation EGFR TKIs seem to bring new hopes for patients who progressed after treated with EGFR TKIs. And the T790M mutation-positive patients will be obtained greater efficacy than T790M mutation-negative patients. Recently, more and more clinical trials put three-generation EGFR TKIs into first-line treatment for NSCLC. Ongoing trials will explore whether the patient with intrinsic resistance NSCLC can benefit from the third-generation EGFR TKIs [50, 51]. Regrettably, there are new resistance mechanism to challenge third-generation EGFR TKIs, such as HER2 amplification, CMET amplification, KRAS G12S mutation, histological transformation, and EGFR L718Q mutation. Among these, C797S mutation was considered to be the biggest difficulty to osimertinib [52, 53]. Therefore, it is urged to develop new agents to target this particular mutant.

### Fourth-generation EGFR TKIs

The EGFR C797S mutation, occurring in 32% of patients, can keep irreversible EGFR TKIs from covalent binding [54–56]. In order to overcome the new mutation, the fourth-generation EGFR-TKIs appeared.

#### EAI045

EAI045 is the first allosteric inhibitor that targets T790M and C797S EGFR mutants. Interestingly, it is only when combined with cetuximab that EAI045 is working. The investigators found that EAI045 was markedly more active in dimerization-defective EGFR mutants. EAI045 apparently inhibited the proliferation of Ba/F3 cells bearing L858R/T790M mutation when combined with cetuximab that can block EGFR dimerization through preventing EGF ligand binding [57].

The emergence of EAI045 has brought a glimmer of hope for NSCLC patients, but the current studies are only in a preclinical stage. Besides, because the C797S mutation is not the only mechanism for resistance to third-generation EGFR TKIs, EAI045 did not completely overcome the problems of those resistances. So there is still a long way to go for fourth-generation EGFR TKIs.

### EGFR TKI versus EGFR TKI

After more than decades of research, the market has appeared several generations of EGFR-TKI. At present, the most specific and urgent problem is to choose the right EGFR-TKI to maximize clinical benefits for patients, which is also the significance of trials to compare with different drugs. As shown in Table 3.

**Table 3:** Randomized studies of comparing the EGFR TKI in advanced NSCLC with EGFR mutations patients

Study	EGFR TKI	EGFR TKI	Median PFS (months)	ORR (%)	Median OS (months)
CTONG 0901	Gefitinib	Erlotinib	10.4 vs 13.0	52.3 vs 56.3	20.1 vs 22.9
LUX-Lung 7	Afatinib	Gefitinib	11.0 vs 10.9	70 vs 56	27.9 vs 24.5
ARCHER 1050 (NCT01774721)	Dacomitinib	Gefitinib	NA	NA	NA
NCT02296125	AZD9291	Gefitinib or Erlotinib	NA	NA	NA
NCT02186301	Rociletinib	Erlotinib	NA	NA	NA

PFS = Progression Free Survival.

ORR = Objective Response Rate.

OS = Overall Survival.

NA = Not Available.

#### Gefitinib VS Erlotinib

CTONG0901 trial [58] was the primary prospective head-to-head study to compare the first-generation EGFR-TKIs in advanced NSCLC with EGFR mutations patients all over the world. 256 eligible patients were assigned to randomly receive erlotinib or gefitinib until disease progression or unacceptable toxicity. However, the primary end point didn’t reached (mPFS gefitinib vs erlotinib, 10.4 VS 13 m, HR 0.81, *P* = 0.108). Besides, regardless of the EGFR exon19 or exon 21 mutations, erlotinib did not receive a significantly better efficiency and survival benefits than gefitinib. Both of them had similar toxicity. Meanwhile, no matter erlotinib arm or gefitinib arm, patients with EGFR exon19 had significantly better RR and OS than those with exon21 mutations.

#### Afatinib VS Gefitinib

LUX Lung-7 [59] was the first randomized study that head-to-head to compare first-generation and second-generation EGFR-TKI. In this study, 319 NSCLC patients with common EGFR mutation-positive were randomized to either afatinib or gefitinib in first-line. Finally, afatinib significantly improved PFS (11.0 vs. 10.9 months, *P* = 0.017) and TTF (13.7 vs. 11.5 months, *P* = 0.0073) of patients than gefitinib. Both the risk of progression and treatment failure was reduced by 27% and the results were consistent across subgroups including EGFR mutational subgroups. AEs in both groups were consistent with previous experience (drug-related AE, afatinib = 98.8%, gefitinib = 100.0%), and were manageable leading to equally low rates of treatment discontinuation (6.3% for both treatment groups). After a median follow-up of 42.6 months, the median OS was no statistically significant difference between afatinib group and gefitinib group (median OS, 27.9 versus 24.5 months; HR, 0.86; 95% CI 0.66–1.12; *P* = 0.2580) [60].

For real-world clinical practice and treatment guidelines, the 0.1-month PFS benefit do not have too much influential. However, this result may indicate afatinib has broader and longer-lasting inhibitory effect and is capable to extend the time of response in comparison with gefitinib. For instance, afatinib can inhibit signal transduction of ErbB2 and ErbB3, which are associated with acquired tolerance to first-generation EGFR TKIs. Additionally, previous study reflected that afatinib is effective to target EGFR with the T790M mutation, and preceding studies have demonstrated that afatinib has potential efficacy to benefit patients with progression after first-generation TKIs [59].

#### Dacomitinib VS Gefitinib

Nowadays, a phase 3 study to evaluate dacomitinib versus gefitinib at first-line treatment for advanced NSCLC is underway(ARCHER 1050). Investigators randomized 440 patients to dacomitinib (45 mg daily) or gefitinib. (250 mg daily) in a 1:1 ratio. This study is expected to yield preliminary results in 2017. (ClinicalTrials.gov Identifier: NCT01774721).

#### Osimertinib VS Gefitinib or Erlotinib

A phased III study is underway to compare osimertinib with gefitinib or erlotinib as first-line treatment for patients with locally advanced or metastatic NSCLC. PFS is the primary end point, and assessment of PFS is the secondary end points which based on T790M mutation status before treatment and the common EGFR mutation (exon 19 deletion or L858R) tested by circulating tumor DNA. We are expecting early clinical results of this experiment. (NCT02296125).

#### Combined therapy

EGFR TKI monotherapy brought obvious but limit benefits for NSCLC with EGFR mutations, so several clinical trials of combined therapy based on EGFR TKI are being investigated, and many of these have achieved satisfactory results.

### EGFR TKI plus chemotherapy

NEJ005/TCOG0902 [61] is the earliest randomized study to evaluate the association efficacy and safety of EGFR-TKI and chemotherapy in the EGFR mutation-positive patients. The investigators randomized 80 untreated NSCLC patients with EGFR mutation to either a concurrent or a sequential treatment plan with gefitinib and classic chemotherapy(carboplatin/pemetrexed). The final results showed that the mPFS was 18.3 months (*n* = 41) and 15.3 months (*n* = 39) in the concurrent and sequential alternating regimen groups, respectively[HR 0.71 (0.42–1.20), *P* = 0.20]. Preliminary results reported that the median OS got a significant result: 41.9 versus 30.7 months, respectively [HR 0.51 (0.26–0.99); *P* = 0.042]. Besides, both groups got similar response rates (87.8% and 84.6%). The adverse events commonly observed were reversible, and no one fatal interstitial lung disease appeared. Before this final analysis, the new phase III NEJ009 (UMIN000006340) study has begun and now is no longer recruiting, and the purpose of this trial is to compare standard gefitinib with or without chemotherapy in the EGFR-mutated individuals.

FAST-ACT II [62] is the first randomized phase 3 study to demonstrate the efficacy of intercalated regimen of chemotherapy in combination with an EGFR inhibitor for patients with advanced NSCLC. Patients untreated previously with advanced non-small cell lung cancer randomly received six cycles of chemotherapy cooperation with intercalated erlotinib or placebo orally every 4 weeks. Based on the final results, it can be found that PFS was significantly better in the combined erlotinib arms than placebo arms ( mPFS 7·6 vs 6·0 months, HR 0·57 [0·47–0·69]; *p* < 0·0001). In addition, the median OS also supported the former (18·3 months and 15·2 months, respectively; HR 0·79 [0·64–0·99]; *p* = 0·0420). Besides, in patients with EGFR mutation-positive, the benefits of treatment were significant difference (mPFS 16·8 vs 6·9 months, HR 0·25 [0·16–0·39]; *p* < 0·0001; mOS 31·4 months [22·2–undefined] vs 20·6 months [14·2–26·9], HR 0·48 [0·27–0·84]; *p* = 0·0092). In terms of safety, slightly more patients in placebo group were reported to have serious adverse events (76 (34%) of 222 versus 69 (31%) of 226 ), and the commonly observed grade 3 or greater adverse events were neutropenia, thrombocytopenia, and anaemia.

A randomized phase II trial (JMIT) [63] investigated gefitinib combined with pemetrexed and gefitinib alone as first-line therapy for advanced nonsquamous NSCLC with activating EGFR mutations. Investigator randomized 195 Asian patients with EGFR mutations to pemetrexed plus gefitinib or gefitinib alone in 2:1 ratio. The median PFS was 15.8 months in P+G set, which was significantly longer than 10.9 months in gefitinib set (adjusted HR, 0.68; 95% CI, 0.48 to 0.96; one-sided *P* = .014; two-sided *P* = .029).The results of progressive disease and duration of response were consistent with mPFS (median, 16.2 v 10.9 months, respectively; HR, 0.66; 95% CI, 0.47 to 0.93;median, 15.4 v 11.3 months, respectively; HR, 0.74; 95% CI, 0.50 to 1.08). Moreover, ORR were similar and the overall survival data are immature between two groups. In safety, the combination group had more grade 3 or worse AEs, but toxicities were common and manageable.

### EGFR TKI plus anti-angiogenic drugs

Admittedly, angiogenesis is necessary for tumor development and invasion, so a high microvessel density has been identified as a predictive factor of metastasis [64]. VEGF is regarded as the most potent regulator of angiogenesis and is the target of NSCLC verification [65, 66]. The VEGF and EGFR pathways are known to be interrelated. Activation of EGFR can increase VEGF expression by its ligands at the cellular level [64, 67].Similarly, EGFR inhibition can make VEGF down-regulated through both hypoxia-inducible factor-α-dependent and independent mechanisms [68–74].

Some preclinical studies intimated the anti-angiogenic monoclonal antibody bevacizumab could enhance antitumor activity when used in combination with EGFR-TKI in NSCLC cells carrying an EGFR mutation, especially in cells that express high levels of VEGF [64].

In several trials, combining the anti-angiogenic monoclonal antibody bevacizumab with EGFR-TKI has exhibited additional efficacy in unselected NSCLC patients [75, 76]. In a phase 3 BeTa study that compared the combination of erlotinib and bevacizumab with erlotinib alone as second-line strategy for NSCLC. The subgroup analysis of EGFR mutation-positive participants reported that mPFS in the combination group was substantially longer than monotherapy group (17·1 months vs 9·7 months). [76, 77] According to this study, Takashi Seto’*el* undertook an randomized, phase 2 trial (JO25567) [64, 78] to examine the efficacy and safety of erlotinib with or without bevacizumab as first-line therapy for patients with EGFR mutation NSCLC. Finally, this study presented that the PFS of combination therapy was longer than that of erlotinib monotherapy (16·0 months and 9·7 months, hazard ratio 0·54, 95% CI 0·36–0·79; *p* = 0·0015). In addition, the incidence of serious adverse events was equivalent in both groups (18 [24%] patients in two drug group and 19 [25%] patients in single drug group). Grade 3 or worse AEs observed were rash, hypertension, and proteinuria.

The results of BELIEF study have exhibited in the 2015 European Cancer Congress,and it showed that the ORR (76.1%) and PFS (13.8 months; 95% CI: 10.3–21.3) of combination with bevacizumab and erlotinib were significantly improved independent of T790M status. The 1-year PFS of T790M mutation positive was 72.4% and median PFS was 16.0 months (95% CI: 13.1-NE). By contrast, the data were 49.4% and 10.5 months (95% CI: 9.2–16.2) in patients without T790M mutation. The safety trial of osimertinib combined with bevacizumab for advanced NSCLC with EGFR mutations is recruiting. (NCT02803203).

The above studies show that the combination of bevacizumab and EGFR TKIs may be a promising pattern for EGFR mutant patients, even for those with intrinsic resistance to EGFR TKIs. The combination mechanism of bevacizumab may enhance the anti-tumor activity of EGFR TKIs and perhaps increase intratumoral concentration of EGFR TKIs to partially reverse intrinsic resistance [79].

### EGFR TKI plus immunotherapy

The PD-1/PD-L1 checkpoint inhibitors have brought an promising prospect in advanced NSCLC, such as nivolumab and pembrolizumab [80–82]. Based on this reason, they have been already recommended to apply to clinical treatment in advanced NSCLC.

High expression of PD-L1 is an efficacy predictor of PD-L1 inhibitors and prognostic marker in tumor tissues in non-squamous NSCLC [81]. Interestingly, the expression of PD-L1 is associated with EGFR mutation. It is reported that the NSCLC cell lines with activating EGFR mutations expressed higher PD-L1. That is to say, EGFR mutations contribute to the up-regulation of PD-L1 expression [83–87]. As part of TATTON study, the combined application of osimertinib and durvalumb in NSCLC patients harboring EGFR mutation was investigated. 11 EGFR TKI-naive patients (Part B, dose expansion) were treated with 80 mg osimertinib plus 3 mg/kg or 10 mg/kg durvalumab every fortnight. The results showed that ORR was 70%. However, this combination was associated with high incidence of interstitial lung disease (ILD), which was noted in 64% [7/11]. So far, the underlying mechanism associated with high incidence of ILD is still unknown and being investigated. Thus, further enrollment of this arm has been permanently suspended [88]. Another combination of gefitinib plus durvalumab is being investigated in ongoing phase Ib study [89]. In dose-expansion phase, 10 EGFR TKI-naive patients were treated with concurrent durvalumab (10 mg/kg every fortnight) and gefitinib (250 mg qd) in arm 1 or treated with gefitinib monotherapy lead-in for 4 weeks followed by concurrent durvalumab plus gefitinib in arm 2, respectively. The ORR was 77.8% in the former, and 80.0% in the latter, respectively. And the more common treatment-related AEs were demonstrated in 80% and 60% for each arm. However, the adverse of grade 3/4 were alanine aminotransferase (ALT) elevation (70% in arm 1vs. 60% in arm 2) and aspartate aminotransferase (AST) elevation (40% in arm 1 vs. 50% in arm 2) in both arms. Currently, there are several clinical studies in progress now and some important data will soon be published about the combination of EGFR TKIs and immunotherapy, such as GEFTREM, NCT02630186 and NCT02496663.

Combined therapy based on EGFR TKI with better efficacy and controllable adverse effects (Table 4) could be applied to clinical treatment as a potentially valuable treatment regimen. However, not all combined therapy models can be applied. Clinicians should choose the most appropriate treatment for patients based on patients’ performance status, economic status, personal wishes, and clinical experience.

**Table 4:** Adverse effects of combined therapy in major clinical trials

Study	Neutropenia	Anemia	Thrombocytopenia	Diarrhea	Rash	Paronychia	Stomatitis	AST/ALT elevation	LD	Hypertension
	≥ Grade3 (%)	≥ Grade3 (%)	≥ Grade3 (%)	≥ Grade3 (%)	≥ Grade3 (%)	≥ Grade3 (%)	≥ Grade3 (%)	≥ Grade3 (%)	≥ Grade3 (%)	≥ Grade3 (%)
NEJ 005^1, 2^	48.8	34.1	41.5	9.8	2.4	2.4	4.9	9.8	0.0	NA
	46.2	12.8	28.2	0.0	0.0	2.6	0.0	20.5	NAa	NA
FASTACT-2^3^	29	11	14	1	5	NA	0.0	NA	NAb	NA
JMIT^4^	5	3	NA	1	NA	NA	4	22	1	NA
JO25567^5^	NA	NA	NA	1	25	3	1	NAc	0	60

^1^Gefitinib + chemotherapy Concurrent regimen.

^2^Gefitinib + chemotherapy Sequential alternating regimen.

^3^Erlotinib + chemotherapy.

^4^Gefitinib + Pemetrexed.

^5^Erlotinib + Bevacizumab.

AST = Aspartate Aminotransferase.

ALT = Alanine AminotransferaseT.

ILD = Interstitial Lung Disease.

NA = Not Available

^a^A total of 4 interstitial lung diseases (5% of all patients) occurred (grade 1 and 2 events in the concurrent, and grade 2 and 4 events in the sequential alternating regimen group).

^b^One interstitial lung disease event occurred.

^C^5% incidences of liver function disorder or abnormal hepatic function were noted.

### Future challenges

#### Intratumor heterogeneity

Although EGFR TKI can benefit patients with EGFR mutation, drug resistance is still unavoidable, Intratumor heterogeneity may be the lead culprit of cancer resistance, and introduce significant challenges in designing effective treatment strategies. It is reported that the cancer stem cell model and the clonal evolution model were served to explain the heterogeneity of tumour cells [90–92]. We believe that these patterns are coexistent, and they are both conducive to heterogeneity in different stages, such as tumour growth and disease aggression. We found that the use of cytotoxic drugs usually lead original tumor shrinkage. This means the disruption of the original non-resistant subclonal populations in heterogeneous tumors, leaving only resistant clones. After a period of time, these resistant tumour populations could replicate and develop a new tumour by the branching evolution mechanism. The new tumour may be heterogeneous and insensitive to the used therapy, even more aggressive. TRACERx study showed that intratumor heterogeneity can increase the risk of recurrence of tumor and death of patients by chromosome instability. As a result, chromosome instability may be a potential prognostic predictor [93–94]. And up to now, there are still no effective predictors to evaluate treatment efficacy and progress tendency. Therefore, the more understanding about heterogeneity can provide a deeper recognization to the occurrence and development of tumor. Conversely, it is important to guide precision medicine strategies that integrate knowledge of heterogeneity to achieve higher efficacy.

The detection of tumour heterogeneity and resistance mechanisms after EGFR TKI therapy largely relied on tumor biopsies. TRACERx [93] study found that 86% tumor regions carried only a single branch of the phylogenetic tree in a median of 5 per tumor (range, 2 to 15). If using a single diagnostic biopsy sample, other information about the tumor would be missed, and lead to a mixed response in subsequent treatment. Therefore, multi-point sampling, dynamic monitoring can be fully reflected evolution process of tumor from early stages. It was reported that ctDNA can prompt the recurrence time of tumor ahead 70 days, than the traditional imaging examination [95]. A study that compares 1,033 tissue samples with 803 plasma samples from the same patients found that the concordance between ctDNA analysis and tissue at diagnosis was 94.3% in identifying EGFR mutations, with 65.7% sensitivity and 99.8% specificity [96]. Besides, the T790M mutation could be also detected effectively through using plasma DNA [97–99].However, a study about early stage lung cancer showed that histological subtype influenced ctDNA detection: 19% (11/58) of lung adenocarcinomas were ctDNA positive, compared with 97% (30/31) of lung squamous cell carcinomas. Furthermore, driver event in EGFR was not associated with ctDNA detection within lung adenocarcinoma [95]. It indicates that ctDNA still has huge challenges when facing lung adenocarcinoma with EGFR mutation. In addition,, aurine-based test, RT-PCR and digital PCR assays are also in progress. Using plasma-based molecular testing to monitor disease status and clinical response to therapy may help to make decisions more rapidly.

### Unknown EGFR mutations

It is known that all patients with mutant EGFR will ultimately generate resistance to EGFR TKI therapy after first or secondary generation EGFR TKI. Drugs targeting these mutations have been published, such as third-generation EGFR TKIs. Now, four-generation EGFR TKIs targeting three-generation drug resistance drugs are also on the road. Then what? Because the mechanism of drug resistance is not yet clear, targeted therapy of lung cancer would fall into the dilemma of drug resistance again. If the three-generation EGFR TKIs were applied to the front line, we don’t know whether it will produce similar to the effect of “multiple drug-resistant bacteria”, making the follow-up treatment of NSCLC insensitive. Therefore, in order to determine more unknown mutations, the relevant research should closely monitor the response to subsequent treatment of tumor.

### Detecting technologies in ctDNA

CtDNA detection has been commonly used in clinical practice to detect EGFR mutation state besides tissue sampling [100–101]. Thus, for the accuracy and convenience of clinical application, it is necessary to have a higher level technique for detection in ctDNA. The more popular detection strategies for plasma EGFR analysis are amplification refractory mutation system (ARMS), droplet digital PCR (ddPCR) and next-generation sequencing (NGS)-based methods [102]. Among them, ADx-ARMS (ADx^®^ EGFR 29 Mutations Detection Kit) and Cobas-ARMS (cobas^®^ EGFR Mutation Test v2) have been approved for clinical use by the China Federal Drug Administration (CFDA) and the U. S. Food and Drug Administration (FDA) for the detection of EGFR mutations in plasma respectively. By comparison, ddPCR and NGS-based methods are extensively applied in study because of their quantitative advantage. However, the lasted study confirmed the capacity of four major detection systems to detect EGFR mutations in ctDNA from a small sample and the results indicated that three methods except ADx-ARMS all showed better sensitivity. When the allele frequency of EGFR mutations is more than 1% in plasma and tissue samples, ADx-ARMS may be a better choice as a qualitative detection by comparison [102]. In general, Firefly NGS platform may be the most sensitive technique for EGFR mutational profiling considering the sensitivity, specificity, mutation information, and the clinical efficacy of EGFR [102]. However, because the sample size is too small, there will be a larger prospective study to validate those findings. What we needed is a more accurate, less invasive diagnostic approach, which provides us the right directions in future researches.
